# 3D Dirac semimetals supported tunable terahertz BIC metamaterials

**DOI:** 10.1515/nanoph-2022-0285

**Published:** 2022-10-21

**Authors:** Xiaoyong He, Fangting Lin, Feng Liu, Wangzhou Shi

**Affiliations:** Department of Physics, Mathematics & Science College, Shanghai Normal University, No. 100 Guilin Road, Shanghai, 200234, China; Shanghai Key Lab for Astrophysics, No. 100 Guilin Road, Shanghai 200234, China

**Keywords:** BIC resonance, Dirac semimetals, metamaterials, terahertz

## Abstract

Based on the 3D Dirac semimetals (DSM) supported tilted double elliptical resonators, the tunable propagation properties of quasi-bound in continuum (BIC) resonance have been investigated in the THz regime, including the effects of rotation angles, DSM Fermi level, and the configuration of resonators. The results manifest that by altering the rotation angle of elliptical resonator, an obvious sharp BIC transmission dip is observed with the *Q*-factor of more than 60. The DSM Fermi level affects the BIC resonance significantly, a sharp resonant dip is observed if Fermi level is larger than 0.05 eV, resulting from the contributions of reflection and absorption. If Fermi level changes in the range of 0.01–0.15 eV, the amplitude and frequency modulation depths are 92.75 and 44.99%, respectively. Additionally, with the modified configurations of elliptical resonators, *e.g.* inserting a dielectric hole into the elliptical resonator, another transmission dip resonance is excited and indicates a red shift with the increase of the permittivity of the dielectric filling material. The results are very helpful to understand the mechanisms of DSM plasmonic structures and develop novel tunable THz devices, such as modulators, filters, and sensors in the future.

## 1 Introduction

In recent years, terahertz (THz) waves witness the innovative applications in the fields of astronomical observation, medical imaging, homeland security identification, and high-speed 6G wireless communication [1–6]. For instance, broadband THz emission via surface optical rectification from a 19 nm thin layer of indium tin oxide was demonstrated, due to the focus of the pump laser fields related with the epsilon-near-zero effect, the generated THz signal was enhanced significantly and overcame the restriction of the phase-matching condition, its bandwidth was over 3 THz [4]. But to the further substantial developments of THz science and technology, it is highly demanded to explore functional devices and components with fine performances. Artificially made of well-ordered subwavelength resonators, metasurfaces are capable of effectively regulating the electro-magnetic properties of incident waves [7–12]. However, the further exploration of metasurfaces devices to control the light in an arbitrary desirable manner is hindered by the large radiation losses and poor electrical tunability. Novel emerging materials, such as black phosphorus, transition metal molybdenum disulfide, graphene, and topological materials, provide good platforms for the exploration flexible functional devices [13–20]. As an important type of topological materials, Dirac semimetals (DSM) manifests the merits of linear dispersion, large fermion degeneracy, and especially the dynamical manipulation of conductivity, which sheds new possibilities design of THz devices [21–26].

Originally proposed by von Neuman and Wigner in 1929, bound states in the continuum (BICs), *i.e.* a perfected non-radiative discrete bound state coexisted within a continuum spectrum of spatially extended states, is formed through destructive interference between leaky modes and shows an infinite *Q*-factor [27–30]. However, thanks to the fabrication imperfections, roughness, and material loss, a quasi-BIC resonance with sharp peak and finite *Q*-factor appears [31–34]. Inhibiting the peculiar ability of full suppression of radiation losses and strongly confined modes, the quasi-BICs in metasurfaces structures are closely associated with Fano resonances, trapped modes, and plasmon induced transparency [35–38], which attracts the attentions of many researchers. For instance, by utilizing pairs of tilted Si nano-bars F. Yesilkoy *et al.* investigated the quasi-BIC phenomenon in the near-IR spectral region, the *Q*-factor was about 144, near-field enhancement was increased by 40 times, and the refractometric sensitivity reached about 263 nm per refractive index unit [39]. Based on a hybrid structure of uniform graphene membrane and Si nanodisks, X. Wang *et al.* showed due to the enhancing interaction between the radiation engineering and BIC, the absorption bandwidth was modulated more than two orders of magnitude, *i.e.* 0.9–94 nm, by changing the asymmetric parameter of metasurfaces, the Fermi level, and layer number of graphene [40]. By covering part section of metal split ring metasurface with a thin Ge layer of thickness about 500 nm, a dynamically controllable quasi-BIC resonance was excited in the THz region, 200% transmission intensity modulation of the quasi-BIC resonance was achieved by photo-excited the Ge stripe, and the recovery time was within 7 ps [41]. With a thin MgF_2_ layer inserting into periodic Si nano-pillars arranged in square lattices and Ag substrate, a hybrid dielectric-metal supporting symmetry protected Friedrich–Wintgen BIC resonance was demonstrated, which increased the lifetime of optical mode and minimized the mode volume simultaneously, the electric field was strongly confined in the dielectric particles and reduced the mode volume one order of magnitude [42].

It is crucial to develop flexible, low-cost, and high efficient THz devices with simple fabrication methods. The performances of MMs structure are closely associated with the configuration of unit cells, such as tilted resonators, inserting a hole in the resonator or adopting the hybrid structures [43, 44]. For noble metal MMs resonators, the *Q*-factor of resonant curve is not very large, and the operating wavelength is designed at a fixed value. Similar to graphene layer, 3D DSM layer inhibits strong light confinement, low dissipation, and good tunable conductivity. Furthermore, 3D DSM has also several advantages, such as the higher Fermi velocity and mobility, surmounting the restriction of thickness and an additional structural degree-of-freedoms in the construction of functional devices [17–20, 44–46]. It is widely expected DSM is good platform to design novel flexible functional devices. To explore high efficient tunable THz devices, the tilted elliptical DSM MMs have been investigated, indicating an obvious sharp BIC transmission dip with the *Q*–factor of more than 50. The DSM Fermi level affects the resonant curve significantly, sharp resonant curve is achieved if Fermi level is larger than 0.05 eV, the amplitude and frequency modulation depth (MD) are 92.75 and 44.99%, respectively. Additionally, with the modified configuration of elliptical resonators (such as the hetero-structure hybrid resonators or dielectric filling materials in the DSM resonators), another transmission resonant dip is observed.

## 2 Structural design and research methods

**Figure 1:**
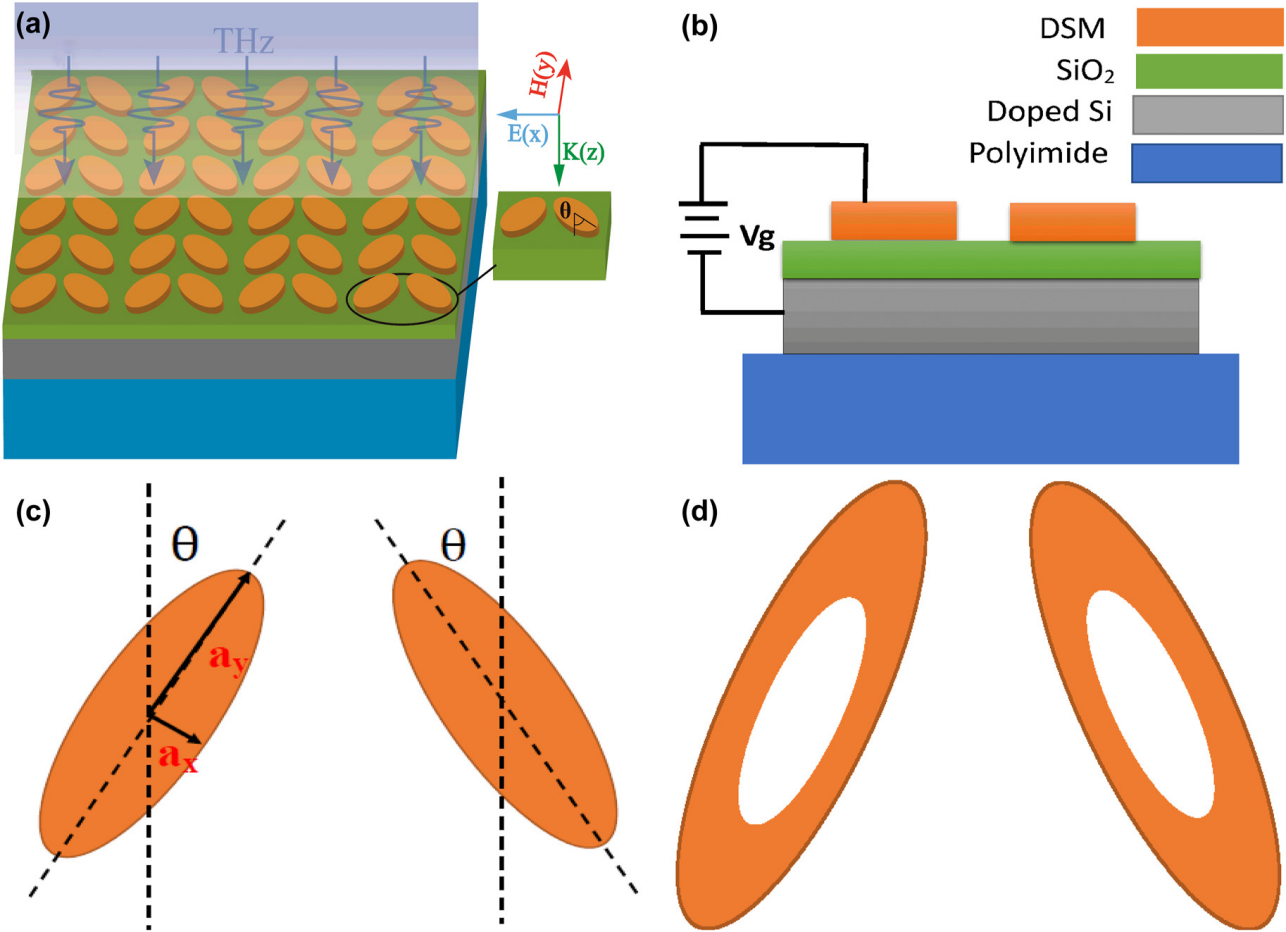
The sketch of the proposed DSM double elliptical MMs structures. (a) The 3D geometry configuration of the DSM double elliptical MMs structure. (b) The side view and (c) top view of the DSM supported elliptical BIC MMs structure. (c) The top view of tilted DSM sketch structure. (d) The top view of hollow DSM resonators. The DSM MMs structures are deposited on the SiO_2_/Si layers, the thickness of SiO_2_ layer is 30 nm, and the doped Si layer is utilized to apply the gate voltage with a thickness of 2 μm. The length of semi-axis along the *x* and *y* directions are 60 and 7.5 μm, respectively. The period length along the *x* and *y* directions are both 128 μm.

Figure 1 illustrates the geometry configurations of DSM supported BIC elliptical MMs structure, which are situated on the SiO_2_–Si–polyimide multilayers. The thickness of polyimide substrate layer is 2 μm. The rotation angle of double elliptical resonator is *θ*; the polarization direction is along the *x* direction. THz waves incidents on the MMs structure along the *z* direction.

Under the framework of Kubo formalism in random phase approximation, the longitudinal complex dynamic conductivity of the 3D DSM can be expressed as [47]:

(1) $$Re\sigma_{\text{DS}}\left(\Omega \right)=\frac{e^{2}}{\hbar }\frac{gk_{F}}{24\pi }\Omega G\left(\Omega /2\right)$$

(2) $$\begin{array}{ll} Im\sigma_{\text{DS}}\left(\Omega \right) & =\frac{e^{2}}{\hbar }\frac{gk_{F}}{24\pi^{2}}\left[\frac{4}{\Omega }\left(1+\frac{\pi^{2}}{3}{\left(\frac{T}{E_{F}}\right)}^{2}\right)\right.\\ & \;\left.+8\Omega \overset{\varepsilon_{c}}{\underset{0}{\int }}\left(\frac{G\left(\varepsilon \right)-G\left(\Omega /2\right)}{\Omega^{2}-4\varepsilon^{2}}\right)\varepsilon d\varepsilon \right] \end{array}$$

in which *G*(*E*) = *n*(−*E*) − *n*(*E*), *n*(*E*) is the Fermi distribution function, *E*_F_ indicates the Fermi level, *k*_F_ denotes the Fermi wave-vector, **Ω** = ℏ**ω**/*E*_F_, *k*_F_ = *E*_F_/ℏv_F_ represents the Fermi momentum, *v*_F_ is Fermi velocity, *E*_c_ remarks the cutoff energy beyond which the Dirac spectrum is no longer linear, *g* is the degeneracy factor.

The permittivity of 3D Dirac semimetals can be obtained using the following formula,

(3) $$\varepsilon_{\text{DS}}=\varepsilon_{\infty }+i\sigma_{\text{DS}}/\omega \varepsilon_{0}$$

where *ε*_b_ is the effective background dielectric (*ε*_b_ = 1, *g* = 40, for AlCuFe quasi-crystals), *ε*_0_ is the permittivity of vacuum.

The *Q*-factor means the rate of the stored energy and the energy loss in the resonator, which can be expressed as:

(4) $$Q=\frac{f_{\text{res}}}{FWHM}$$

FWHM is the full width at a half maximum of the resonance peak.

To measure the trade-off between *Q*–factor and resonant strength, the figure of merits is defined as following,

(5) $$FOM=Q\times A_{\text{m}},$$

in which *A*_m_ as the amplitude strength of the resonant curve.

## 3 Results and discussion

**Figure 2:**
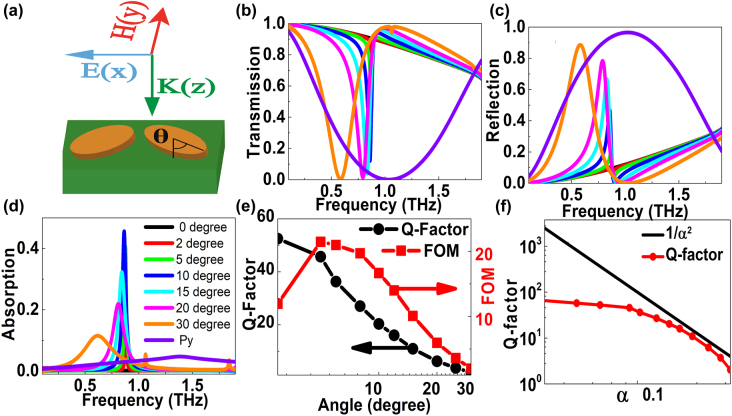
The influences of asymmetric degree on the DSM double elliptical MMs structures. (a) The top view of the elliptical quasi-BIC DSM MMs resonators. (b)–(d) The transmission, reflection, and absorption resonant curves of the tilted elliptical DSM MMs structure at different tilted angles. (e) The *Q*-factors and FOMs of transmission curves versus the tilted angles. The thickness of DSM elliptical MMs is 2 μm. The long and short semi-axes of elliptical MMs are 60 and 7.5 μm, respectively. The period lengths along the *x* and *y* directions are both 128 μm. (f) The *Q*–factor versus asymmetric parameter of BIC transmission resonant dip curves.

We study the BIC resonances based on a symmetry protected quasi-BIC system composed of pairs of the tilted elliptical DSM resonators, as given in Figure 2(a). The orientation of each elliptical resonator is characterized by a rotation angle *θ* between the *y* axis and the long axis of the elliptical bars, thus the asymmetric parameter *α* is defined as sin*θ*. Figures 2(b)–(d) show the transmission, reflection, and absorption curves at different tilted angles. If the polarization is along the *y* direction, the elliptical resonator excites an obvious transmission resonant dip; the reflection curve also indicates an obvious peak, as the violet line given in Figure 2(b), which results from the dipolar resonance of DSM resonators in the THz region. However, if the polarization is along the *x* direction, on the condition that the tilted angle is zero degree, *i.e.* for the symmetric structure, this proposed DSM elliptical structure supports a symmetry-protected BIC and cannot couple to the free space radiation due to symmetry protection. In this case, the DSM resonator cannot excite obvious transmission dip because the semi-axis length along the *x* direction *a*_x_ is small, *i.e.* the black line in the Figure 2(b). If the tilted angle is not zero, a small transmission resonant dip appears. As the tilted angle increases, the resonant strength of transmission curve becomes stronger, the electric inductance increases. Since the resonant frequency are proportional to the 1/(*LC*)^1/2^, the according transmission resonant dip shows a red shift. For instance, if the tilted angles are 2, 5, 10, and 30°, the resonant dip (frequency) is 0.7927 (0.8771 THz), 0.4426 (0.8733 THz), 0.1195 (0.8581 THz), and 0.003460 (0.5845 THz), respectively. The modulation depths of amplitude and frequency are defined as *T*_mod_ = (*T*_max_ – *T*_min_)/*T*_max_ and *f*_mod_ = (*f*_max_ – *f*_min_)/*f*_max_, respectively. Thus, if the tilted angle changes in the range of 2–30°, the amplitude and frequency modulation depths are 99.56 and 33.36%, respectively. The influences of rotation angles on the reflections curves can be found in Figure 2(c). If the value of *θ* is smaller than 5°, the reflection curve peak is not very large. As the rotation angle increases, the BIC phenomenon becomes stronger, the reflection curve indicates a strong peak and obvious red shift, which corresponds with the transmission dip in Figure 2(b). For instance, if the tilted angles are 2, 10, and 30°, the reflection peak (frequency) are 0.1109 (0.8714 THz), 0.4702 (0.8543 THz), and 0.8864 (0.5788 THz), respectively. Thus, the amplitude and frequency modulation depths are 85.86 and 33.58%, respectively. Figure 2(d) shows the absorption curves at different *θ*. The absorption is not very large at small tilted angle, as the value of *θ* increases, the absorption increases significantly. However, if the tilted angle increases further, the absorption decreases. For instance, if the tilted angles are 2, 10, 20, and 30°, the absorption peak (frequency) are 0.1148 (0.879 THz), 0.4565 (0.8638 THz), 0.2192 (0.8087 THz), and 0.1152 (0.613 THz), respectively. The amplitude and frequency modulation depths are 87.48 and 33.58%, respectively. The reasons are given in the following. When the tilted angle *θ* is small, the overall radiative loss is suppressed significantly; a small sharp absorption peak appears, as the red and green lines given in Figure 2(d). As the tilted angle and asymmetric parameter increase, the gap distance between the two elliptical resonator decreases, the interaction between them increases. Thus, much more modes couple into free space, the dissipation increases, resulting in a larger dissipation and broader spectral line width, *i.e.* the magenta and orange lines given in Figure 2. However, if the tilted angle increases further, larger than 10° the elliptical bar length along the polarized *x*-direction increases significantly. The low lossy dipolar resonance plays a dominated role and leading into the dissipation reduction again, as the magenta and orange lines given in Figure 2(d). Therefore, the absorption curves show a peak value at certain tilted angle, about 10°. In a word, at small tilted angle the absorption dominates, the resonant peak position is not sensitive to the tilted angle. On the other hand, if the tilted angle is larger than 10°, the reflection takes an important role and results into sharp BIC transmission resonant dip. The *Q*–factor and FOM can be found in Figure 2(e). At small tilted angle, the overall radiative loss is suppressed significantly, the *Q*–factor is larger than 60. As the tilted angle increases, the *Q*–factor decreases. However, the resonant strength becomes stronger with increase of the value of tilted angle. Thus, the FOM shows a peak at certain angle, about 5–8°, as given in Figure 2(e). Figure 2(f) depicts the effects of asymmetric parameter *α* on the *Q*–factor versus of transmission curves. If the value of *α* is relatively large, >0.1, the symmetry-protected BIC satisfies the inverse-square law 1/*α* ^2^ for relatively large asymmetry parameter *α* > 0.1. However, the inverse square law breaks down at smaller asymmetry, especially *α* < 0.05, which comes from the strong coupling and destructive interference.

**Figure 3:**
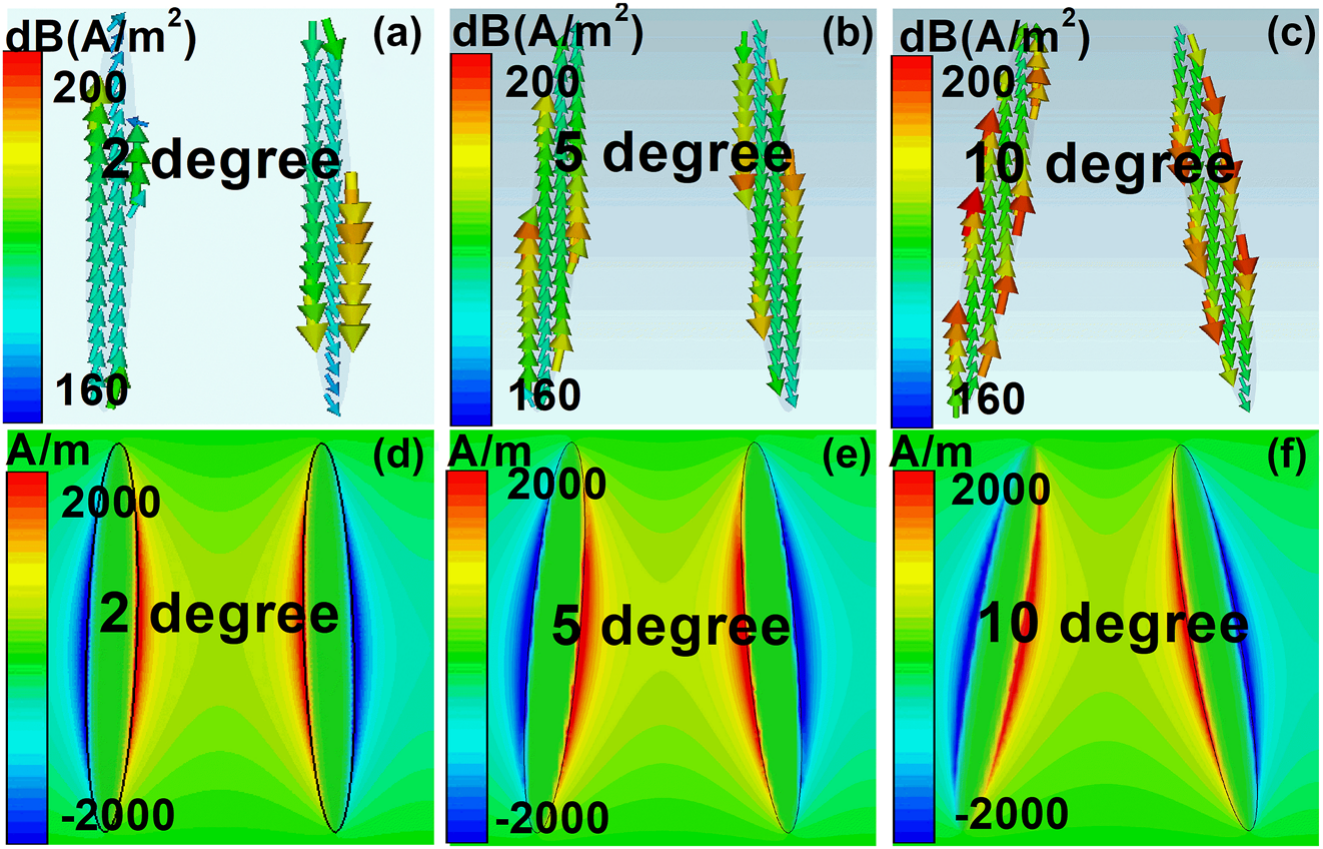
The 2D simulation results of the DSM double elliptical MMs structures at different tilted angles. (a)–(c) The surface current density and (d)–(f) magnetic fields for the elliptical quasi-BIC DSM MMs structures at different tilted angles. The tilted angles are 2, 5 and 10°, and the according resonant frequencies are 0.8790, 0.8752, and 0.8581 THz, respectively. The polarization of the incident light is along the *x* direction.

To have a deep understanding of the tunable mechanisms on the propagation properties, the surface current density and magnetic fields (*H*_z_) of elliptical MMs structures at different tilted angles have been demonstrated in Figure 3. The incident THz waves drive a surface current flowing along the elliptical BIC resonators, and the directions along the left and right resonators are opposite. Thus, a circular loop is formed on the condition that the tilted angle is large, which results in a charge accumulation at gap of the tilted elliptical resonators. The simulation results of magnetic fields can be found on Figure 3(d)–(f). The gap distance between the double DSM elliptical resonators is large at small tilted angle; the interaction between them is weak. As the tilted angle increases, the gap distance reduces, the interaction between resonators increases significantly, the resonant strength becomes stronger. Additionally, since the surface current increases obviously at large tilted angle, the electric inductance enhances as well, which results into the according transmission resonant dip manifests a red shift.

**Figure 4:**
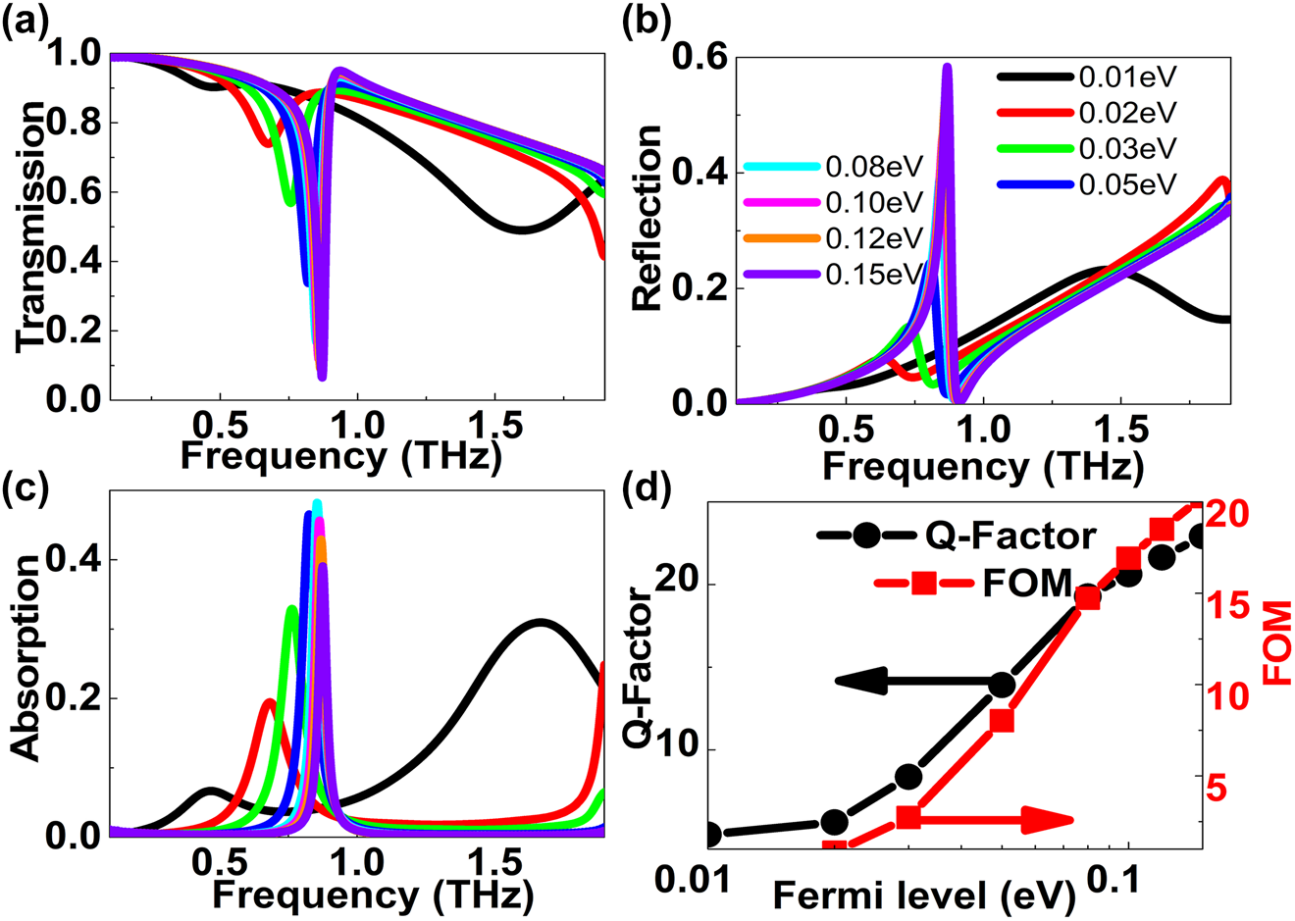
The influences of Fermi levels on the DSM double elliptical MMs structures. (a)–(c) The transmission, reflection, and absorption curves of the elliptical quasi-BIC MMs structures at different Fermi levels. The Fermi levels are 0.01, 0.02, 0.03, 0.05, 0.08, 0.10, 0.12, and 0.15 eV, respectively. (d) The *Q*-factors and FOM of BIC resonance for the transmission curves versus Fermi levels. The rotation angle is 10°.

In analogy to two-dimensional graphene, the complex conductivity and permittivity of 3D DSM can also be dynamically modulated by an applied bias voltage, which can be utilized to manipulate resonant curves efficiently. Figure 4 shows the effects of Fermi levels on the resonant curves. At small Fermi level, *e.g. E*_f_ < 0.02 eV, the tilted elliptical resonator can’t excite obvious BIC transmission dip, as given in Figure 4(a). As Fermi level increases, DSM layer manifests better plasmonic properties, an obvious resonant dip appears. The resonant strength becomes stronger and the resonant dip indicates a blue shift, the BIC resonant dip can be modulated in a wide range. For instance, if Fermi levels are 0.01 eV, 0.05 eV, 0.10 eV, and 0.15 eV, the amplitude (frequency) of resonant dip of high frequency resonances are 0.9030 (0.4762 THz), 0.3378 (0.8201 THz), 0.1195 (0.8581 THz), and 0.06549 (0.8714 THz). Accordingly, the amplitude (frequency) modulation depth is 92.75% (44.99%). The influences of Fermi levels on the reflections curves can be found in Figure 4(b). If the Fermi level is small, <0.05 eV, DSM layer manifests worse plasmonic property, the reflection is weak. As Fermi level increases, the DSM layer shows better plasmonic property, the reflection strength becomes stronger, and resonant peak indicates a blue shift slightly. For instance, at the Fermi levels of 0.02 eV, 0.05 eV, 0.10 eV, and 0.15 eV, the amplitude (frequency) of resonant dips are 0.07864 (0.6339 THz), 0.2444 (0.8068 THz), 0.4702 (0.8543 THz), and 0.5836 (0.8676 THz), respectively. The according amplitude (frequency) modulation depth is 86.53% (26.94%). The absorption curves versus frequency at different tilted angles can be found in Figure 3(c). If the Fermi level is small, the dissipation is weak. As Fermi level increases, the carrier concentration and loss increase. If the Fermi level is about 0.05–0.08 eV, the dissipation reaches a peak 0.5. However, if Fermi level is large enough, *i.e.* >0.10 eV, the reflection dominates, the contribution of absorption decreases. For instance, if Fermi levels are 0.01 eV, 0.05 eV, 0.10 eV, and 0.15 eV, the amplitude (frequency) of resonant dips of high frequency resonances are 0.06669 (0.4686 THz), 0.4654 (0.8258 THz), 0.4565 (0.8638 THz), and 0.3905 (0.8752 THz), respectively. The amplitude (frequency) modulation depth is 92.38% (46.46%). From above discussions, it come to the conclusion that the dissipation dominates at small Fermi level, the transmission dip is not very obvious. With the increase of Fermi level, DSM layer shows better plasmonic properties, the reflection contribution dominates, the loss reduces. The value of *Q*–factor and FOM can be found in Figure 4(d). As Fermi level increases, DSM layer shows better plasmonic properties, the resonant strength of BIC phenomenon becomes stronger, the *Q*-factor and amplitude increases, which results into the FOM enhancing and reaches a peak value of about 20.

**Figure 5:**
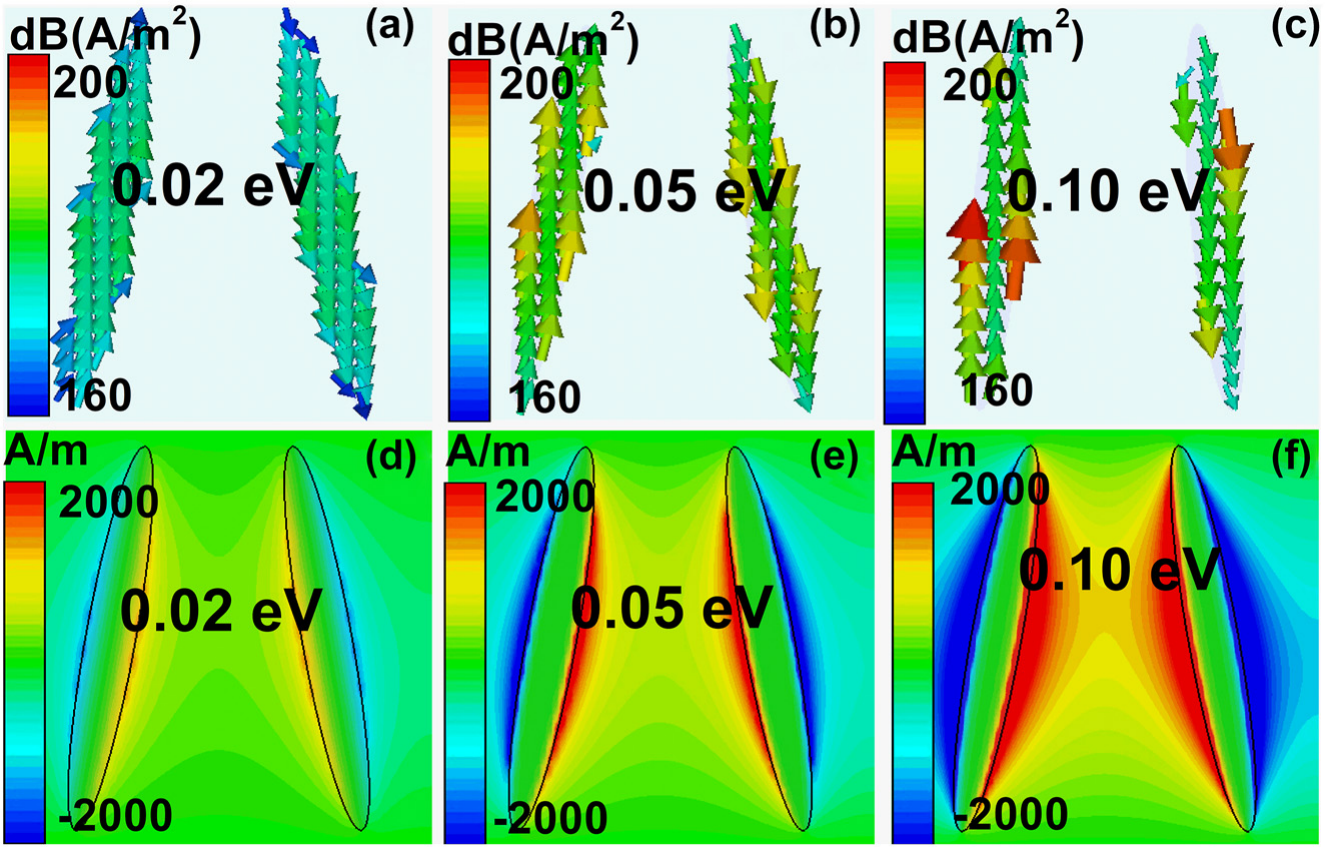
The 2D simulation results of the DSM double elliptical MMs structures at different Fermi levels. (a)–(c) The surface current density and (d)–(f) magnetic fields for the elliptical quasi-BIC DSM MMs structures at different Fermi levels. The resonant frequencies are 0.6738, 0.8201, and 0.8581 THz for the Fermi levels of 0.02, 0.05, and 0.10 eV. The polarization of incident THz wave is along the *x* direction. The tilted angle is 10°.

Figure 5 shows the surface current density and magnetic fields of elliptical MMs structures at different Fermi levels. The polarization is along the *x* direction. The Fermi levels are 0.02 eV, 0.05 eV, and 0.10 eV. From Figure 5(a)–(c), we can find that the surface current flows along the left and right elliptical resonator along different directions. The quasi-BICs boost the electric field enhancement inside metasurfaces. As Fermi level increases, the high permittivity of DSM layer at high Fermi level results in a small skin depth and low material losses. Thus, the resonant strength becomes stronger, and the resonant curve becomes sharper at larger Fermi level. The simulation results of magnetic fields can be found on Figure 5(d)–(f). If the Fermi level is small, the interaction between the left and right elliptical unit cell is weak, the magnetic fields is not stronger. As Fermi level increases, the interaction between the BIC resonators increases because of the better plasmonic properties of DSM layer. If the Fermi level is larger, *e.g.* 0.10 eV, the interaction between the left and right elliptical resonators are very stronger, as given in Figure 5(f).

**Figure 6:**
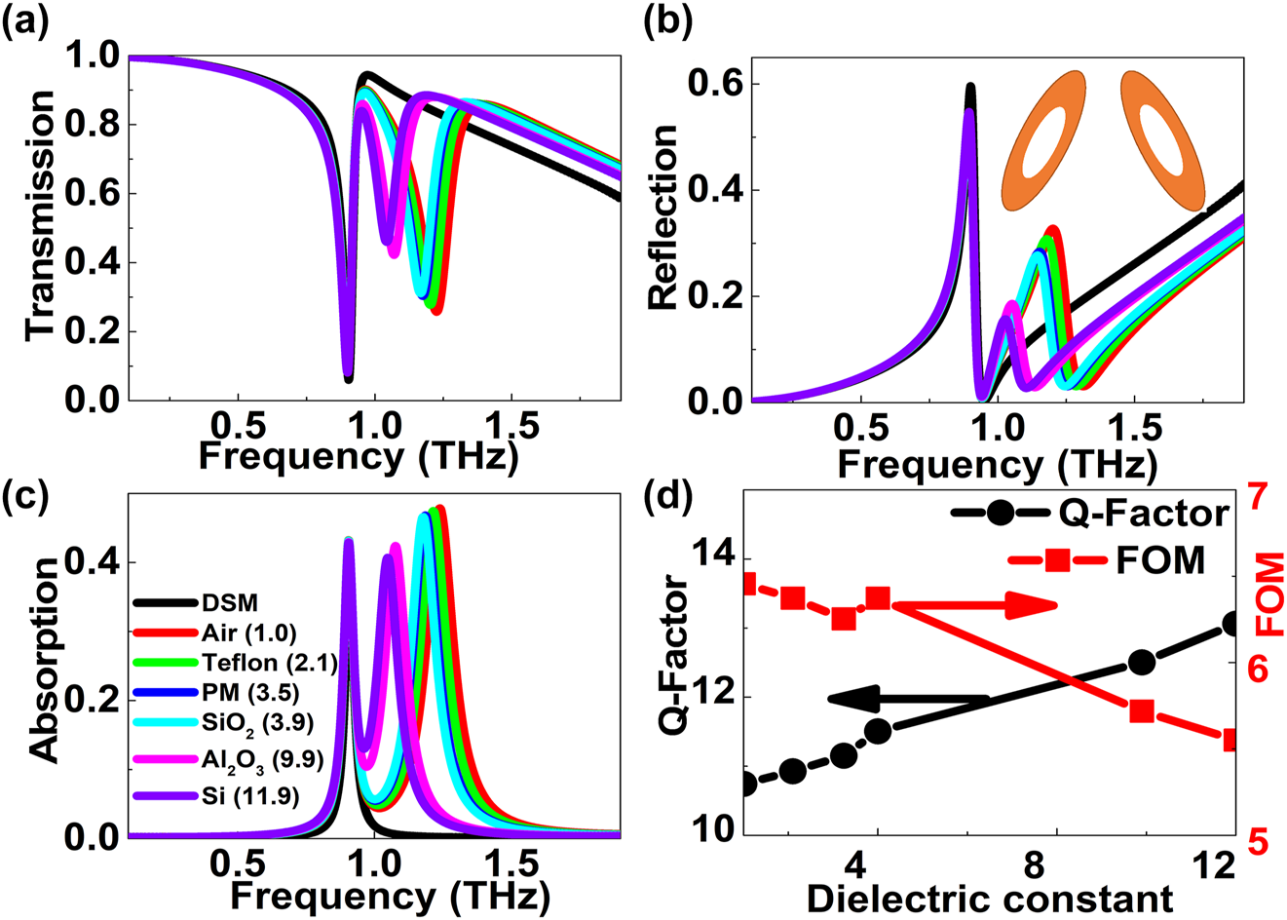
The influences of the different dielectric filling materials on the modified DSM double elliptical MMs structures. (a)–(c) The transmission, reflection, and absorption curves of the modified elliptical BIC MMs structure at different filling materials. (d) The *Q*-factors and FOMs of transmission curves versus different dielectric materials. The tilted angle is 10°. The semi-axes lengths of the elliptical hole in the resonator along the *x* and *y* directions are 40 and 5 μm, respectively.

Figure 6 shows the propagation properties of the DSM modified elliptical BIC structure, *i.e.* a dielectric hole is inserted into the subwavelength resonator, as given in the inset in Figure 6(b). The permittivity of the dielectric filling materials for air, Teflon, polyimide (PM), SiO_2_, Al_2_O_3_, and Si are 1.0, 2.1, 3.5, 3.9, 9.9, and 11.9, respectively [48, 49]. Due to the broken symmetry, another resonant dip at high frequency appears which is significantly affected by the dielectric filling materials. The strength becomes weaker with the increase of the permittivity of dielectric filling material, and the resonant dip also indicates a red shift. For example, if the dielectric filling materials are air, Teflon, polyimide (PM), SiO_2_, and Si, the resonant dip amplitudes (frequencies) are 0.2578 (1.225 THz), 0.2778 (1.202 THz), 0.3035 (1.175 THz), and 0.4604 (1.042 THz), respectively. The reflection curves for the different dielectric filling materials can be found in Figure 6(b). By utilizing the dielectric filling materials with larger permittivity, the reflection peak reduces and the resonant frequency indicates a red shift. For example, when the dielectric filling materials are air, Teflon, SiO_2_, and Si, the resonant dip amplitudes (frequencies) are 0.3276 (1.202 THz), 0.3080 (1.179 THz), 0.2773 (1.145 THz), and 0.1569 (1.027 THz), respectively. The according amplitude (frequency) modulation depth is 52.11% (14.56%). The dissipation curves can be found in Figure 6(c). With the modified hollow elliptical resonator, an absorption peak at high frequency is excited. Furthermore, as the permittivity of dielectric filling material increases, the absorption peak at high frequency enhances and moves to low frequency. From above discussions, it can be found that the high frequency transmission dip is mainly associated with the dielectric materials, the reflection contribution is relatively small, but the dissipation plays an important role. The value of *Q*-factor and FOM can be found in Figure 6(d). As the permittivity of dielectric filling materials increases, the mode confinement improves, the interaction of dielectric filling material with THz waves increases, resulting into the *Q*-factor enhancing. However, since the resonant strength of high frequency resonance becomes smaller, the FOM decreases with the large permittivity of dielectric filling materials, as given in Figure 6(d).

## 4 Conclusions

By depositing the planar arrays of tilted DSM elliptical MMs patterns on the SiO_2_/Si layers, the tunable propagation properties of BIC resonance are given and discussed in the THz regime, taking into accounting the tilted angles, Fermi levels, operation frequencies, and different dielectric filling materials. The results manifest that an obvious sharp BIC transmission dip can be observed, the *Q*-factor and FOM reach more than 60 and 20, respectively. The amplitude and frequency modulation depths of BIC resonance reach more than 99.56 and 33.36% if the tilted angle changes in the scope of 2–30°. The BIC resonant curves are closely associated with DSM Fermi level, the sharper transmission dip can be achieved at larger Fermi level, *e.g.* if Fermi level changes in the range of 0.01–0.15 eV, the amplitude and frequency MD are 92.75 and 44.99%, respectively. If Fermi level is small, the transmission dip results from the absorption, while the contribution of reflection plays an important role if Fermi level is larger than 0.05 eV. Additionally, by introducing a hole in the elliptical DSM structure, another transmission resonant dip is excited at high frequency, which becomes weaker and indicates a red shift with the increase of the permittivity of the dielectric filling material. The results are very helpful to understand the mechanisms of the DSM BIC plasmonic structure and develop novel tunable THz devices, such as modulators, filters, and sensors in the future.
